# Action Choice and Outcome Congruency Independently Affect Intentional Binding and Feeling of Control Judgments

**DOI:** 10.3389/fnhum.2018.00137

**Published:** 2018-04-11

**Authors:** Zeynep Barlas, Stefan Kopp

**Affiliations:** Social Cognitive Systems—Cluster of Excellence Center in Cognitive Interactive Technology (CITEC), Bielefeld University, Bielefeld, Germany

**Keywords:** action choice, outcome congruency, intentional binding, feeling of control, sense of agency

## Abstract

Sense of agency (SoA) refers to the subjective experience that one is in control of their actions and the consequences of these actions. The SoA is a complex phenomenon, influenced by a weighted combination of various prospective (pre-movement) and retrospective (post-movement) processes and factors related to action choice, action selection fluency, action-outcome associations and higher-level inferences. In the current study, we examined the effect of the congruency between actions and outcomes in a context where the choice-level of actions was varied from 1 to 4. The actions consisted of right, left, up and down key presses while the outcomes were visual representations of the actions (i.e., right, left, up and down-pointing arrowheads). Participants performed either an instructed action or freely selected an action among two, three, or four alternatives. Each action randomly produced either a congruent or an incongruent outcome, depending on the matching between the direction of the key press and the direction of the outcome arrowhead. Participants estimated the delay between their actions and the observed outcomes and reported their feeling of control (FoC) over the outcomes. Interval estimations were used as an indirect measure of the SoA to quantify the intentional binding effect, which refers to the perceived temporal attraction between voluntary actions and their outcomes. The results showed that both intentional binding and FoC were enhanced as the choice-level was increased from 1 to 4. Additionally, intentional binding and FoC over the outcomes were stronger when actions produced congruent compared to incongruent outcomes. These results provide additional evidence that both intentional binding and FoC are sensitive to the number of action alternatives and the congruency between actions and their outcomes. Importantly, the current study suggests that these prospective and retrospective cues might independently influence intentional binding and FoC judgments.

## Introduction

Sense of agency (SoA) refers to the subjective experience that one is in control of their actions and the consequences of these actions (Gallagher, 2000; Haggard and Tsakiris, 2009; Haggard and Chambon, 2012). Previous views on the nature of this experience suggested that the SoA can be experienced at two distinct levels. While the high-level SoA is conceptualized through one’s interpretation of their agency, the low-level SoA is experienced non-conceptually and pre-reflective—similar to what we experience in our daily routine of actions (Synofzik et al., 2008a,b).

With respect to the empirical study of the SoA, the high-level SoA can be quantified by the direct measures that are based on the subjective judgments of feeling of control (FoC) over the actions or their corresponding outcomes (Sato and Yasuda, 2005; Linser and Goschke, 2007; Metcalfe and Greene, 2007; Ebert and Wegner, 2010; Wenke et al., 2010; Barlas and Obhi, 2014). Indirect measures, on the other hand, have been introduced as a proxy for the low-level SoA as they do not require conscious reflection on one’s SoA (Synofzik et al., 2008a; Desantis et al., 2011; Moore and Obhi, 2012; Barlas and Obhi, 2014). One such measure is based on the intentional binding phenomenon, which refers to the perceived temporal attraction between voluntary actions and their outcomes (Haggard et al., 2002). More clearly, the temporal interval between actions and outcomes is perceived as shorter when these outcomes are produced by voluntary actions compared to when they follow, for instance, involuntary movements or external causes (Haggard et al., 2002). The strength of this temporal binding of actions and outcomes is considered to index the SoA such that stronger binding is interpreted as stronger SoA (e.g., Engbert et al., 2007; Wenke and Haggard, 2009; Kühn et al., 2012; Moore and Obhi, 2012). It is important to note at this point that the relationship among these measures and their precise relationship with different levels of the SoA remain far from clear (Dewey and Knoblich, 2014; Saito et al., 2015). Nonetheless, relevant research conducted in the last two decades employed these measures extensively to investigate the mechanisms behind the SoA.

An important outcome of the research on the underlying mechanisms of the SoA is that SoA is a complex phenomenon, influenced by a weighted combination of various retrospective (post-movement) and prospective (pre-movement) processes (Moore and Fletcher, 2012; Farrer et al., 2013; Synofzik et al., 2013; Wolpe et al., 2013). Retrospective contributors of the SoA include the higher level inferences based on the observation of actions and outcomes (e.g., Wegner and Wheatley, 1999; Wegner, 2004; Wegner and Sparrow, 2004), causal beliefs (Desantis et al., 2011, 2012b; Haering and Kiesel, 2012) and performance monitoring (Metcalfe and Greene, 2007; Vuorre and Metcalfe, 2016).

A relatively recently proposed prospective cue that has been shown to contribute to the SoA is related to the processes involved in action selection (Chambon et al., 2014; Haggard, 2017; Sidarus et al., 2017b). The major components of action selection processes have thus far been identified as the source of action selection (i.e., self selected vs. externally instructed), the number of action alternatives (i.e., the choice space), and the fluency or the ease of action selection. In one study, for instance, participants could either freely choose to harm or not another participant or they performed the experimenter’s choice of action. Results showed that both binding and electrophysiological brain activity related to the processing of auditory outcomes were attenuated in instructed compared to freely selected actions (Caspar et al., 2016; see also Sebanz and Lackner, 2007; Borhani et al., 2017; Barlas et al., 2018). The effect of action choice has also been observed in the contexts where the number of action alternatives was varied. More specifically, these studies found that both binding (Barlas and Obhi, 2013; Barlas et al., 2017) and FoC ratings (Barlas et al., 2017) are parametrically enhanced as the action choice space was varied from one to several action alternatives. Additionally, numerous studies showed that disfluent or effortful processing of action selection reduced the FoC judgments (Wenke et al., 2010; Chambon and Haggard, 2012; Chambon et al., 2013, 2014; Sidarus et al., 2013, 2017a; Sidarus and Haggard, 2016).

Another important mechanism with both prospective and retrospective components that account for the SoA is based on the comparator model of motor control processes (Frith et al., 2000). According to this model, a copy of the motor command to achieve a specific goal is used by the internal forward models to generate the predictions towards the sensory outcomes of the movement (Wolpert et al., 1995; Wolpert, 1997). Importantly, these predictions are compared to the actual sensory outcomes and, in the case of a discrepancy, one can experience a weaker SoA or attribute the agency to an external agent (Frith et al., 2000; Blakemore et al., 2002; Frith, 2005). Notably, the entire process can be considered to have both a prospective and a retrospective component since the sensorimotor predictions are produced before the movement and the comparison between the predicted and actual outcomes is performed after the movement (Sidarus et al., 2017a).

The role of the matching between predicted and observed outcomes has been examined in various contexts. Sato and Yasuda (2005), showed that discrepancies between previously learned action-outcome associations and actual sensory consequences can reduce the subjective judgment of control. In another study, participants were presented with low or high pitch tones (i.e., auditory primes) before they performed voluntary or involuntary movements (Moore et al., 2009). Critically, the pitch of these primes could be either congruent or incongruent with the actual auditory outcomes, and participants estimated the temporal delay between their movements and the ensuing auditory outcomes. It was found that interval estimations were shorter (i.e., stronger binding) in voluntary than involuntary movements and when the primes were congruent with the outcomes than when they were incongruent. Moreover, the influence of prime compatibility was stronger in the involuntary condition compared to the voluntary condition.

Ebert and Wegner (2010) provided additional evidence for stronger SoA when actions and outcomes were congruent. In their study, participants performed one of two actions (pulling or pushing a joystick) which caused the object on the screen to move either away from or closer to the participant. Participants estimated the delay between their action and the object’s movement as well as their perceived authorship over the object’s movements. The results indicated that both binding and perceived authorship were stronger when the direction of the object’s movement was congruent with the movement of the joystick compared to when it was incongruent. In another study, Farrer et al. (2008) manipulated the sensory feedback of the participants’ movements such that the observed virtual feedback of their movements was modified by varying degrees of spatial perturbation or temporal delay. Participants judged whether the observed movements corresponded to their exact or modified movement or were controlled by the experimenter. The results suggested that at high spatial deviations, participants attributed the observed movements to the experimenter while at medium spatial deviations as well as at medium and high temporal delays they recognized the observed movements as their own but modified movements. These results suggested that spatial and temporal sensory feedback can introduce different effects on the judgment of the source of observed actions.

Other studies, however, showed that the relationship between action-outcome congruency and the SoA—specifically intentional binding—may not be straightforward. In a series of experiments, Haering and Kiesel (2014) examined whether intentional binding depended on the validity of action-effect mappings. In their study, left and right key presses produced congruent outcomes (e.g., red and blue squares, respectively) in 80% of the trials while for the remaining trials, these actions produced incongruent outcomes (Experiment 1). Experiment 2 included a learning session and Experiment 3 rendered the probability of congruent outcomes as %100 and 50% in separate blocks. Overall results suggested that the predictability of the outcomes or the congruency between actions and their outcomes did not influence intentional binding (see also Desantis et al., 2012a).

Overall, above-mentioned studies examining the influence of action-outcome congruency on direct and indirect measures of the SoA suggested that congruent action-outcomes enhance the FoC while the effect of outcome congruency on intentional binding is less clear. In the current study, we aimed to investigate the influence of outcome congruency on both intentional binding and FoC judgments—as the indirect and direct measures of the SoA, respectively—in a context where both action choice-level and action-outcome congruency were manipulated. More clearly, the choice-level in the present study was varied from one to four alternatives (right, left, up and down key presses) and each of these actions could randomly (with a 50% probability) produce a congruent or an incongruent visual outcome. After a left key press, for instance, the congruent outcome would be the display of a left-pointing arrowhead. An incongruent outcome, on the other hand, could be the display of any of the right-, up- or down-pointing arrowheads. The outcomes, therefore, consisted of highly depictive representations of the actions, albeit occurred either congruently or incongruently with the preceding actions. On one view, this could render the relationship between actions and outcomes more “meaningful” (Haering and Kiesel, 2014) similar to the action-effect association used in Ebert and Wegner (2010). We measured both intentional binding using the interval estimation procedure (Engbert et al., 2008; Ebert and Wegner, 2010; Moore and Haggard, 2010; Caspar et al., 2015; Barlas et al., 2017) and FoC judgments over the outcomes.

Based on the previous findings, our prediction towards the effect of choice-level was that both binding and FoC ratings would be systematically enhanced with increased choice-level (Barlas et al., 2017). Regarding the effect of outcome congruency, we expected to find greater FoC when outcomes were congruent with the performed actions compared to when they were incongruent (Sato and Yasuda, 2005; Ebert and Wegner, 2010). The similarity between the nature of the action-outcome relationships (i.e., the perceptual resemblance between actions and outcomes) used in the current study and in the study of Ebert and Wegner (2010) led us to expect stronger binding with congruent compared to incongruent outcomes.

A secondary goal of the current study was to examine whether the prospective signal of choice-level (Barlas and Obhi, 2013; Barlas et al., 2017) and the retrospectively determined outcome congruency would independently affect intentional binding and FoC judgments. The optimal cue integration approach suggests that the SoA is modulated by a weighted combination of prospective and retrospective cues depending on the reliability of each cue present in a specific context (e.g., Moore et al., 2009; Moore and Fletcher, 2012). Based on this view, we conjectured that the effect of choice-level could be stronger when outcomes are incongruent compared to when they are congruent. Additionally, we expected that congruent outcomes would enhance the SoA more strongly when participants perform instructed actions (i.e., one-choice condition) compared to when they freely select among higher number of alternatives.

## Materials and Methods

### Participants

Sample size was determined *a priori* using *GPower 3.1* (Faul et al., 2009), which suggested 22 participants to obtain a significant effect of choice-level (effect size = 0.40 (Barlas et al., 2017), alpha = 0.05, power = 0.95). We recruited slightly more participants as the *a priori* power analyses did not account for the effect of outcome congruency. In total thus, 24 participants from Bielefeld University (14 males, 2 left-handed, *M*_age_ = 24.71 years, *SD* = 2.59 years) took part in the study. Participant exclusion criterion (see below), however, resulted in the exclusion of 2 participants and thus the data of 22 participants (12 males, 2 left-handed, *M*_age_ = 24.73 years, *SD* = 2.60 years) were subjected to the analyses. All participants had normal or corrected-to-normal vision and had no hearing problems. Participants gave their consent prior to the study and received monetary compensation (10 Euros) in exchange for their participation. The study was conducted in accordance with the ethical guidelines of the Declaration of Helsinki and was approved by the Research Ethics Board of Bielefeld University.

### Apparatus and Stimuli

The experiment was developed using PsychoPy (Peirce, 2007, 2008) and run on a Dell personal computer (3.07 GHz). Participants were seated approximately 50 cm away from a 20-inch monitor (resolution: 1600 × 1200) and all stimuli were presented on a white background. Key press responses were made on a 5-key response pad using the left hand’s index finger. On this pad, four keys surrounding the central key were marked with right, left, up and down arrowhead images. An optical wheel mouse was used to indicate responses on the interval estimation and FoC rating scales presented on the screen. The interval estimation scale was ranged from 1 ms to 1000 ms and marked at 50 ms intervals while the FoC scale was a 6-point Likert scale marked at 0.5-point intervals (1: low control; 6: high control). Both scales occupied 1000 pixels on the screen and the horizontal axis position of the mouse cursor after the click (at pixel-precision) was used for the conversion to the corresponding scale value (i.e., between 1–1000 ms for the interval estimation scale and between 1–6 for the FoC rating scale).

**Figure 1:**
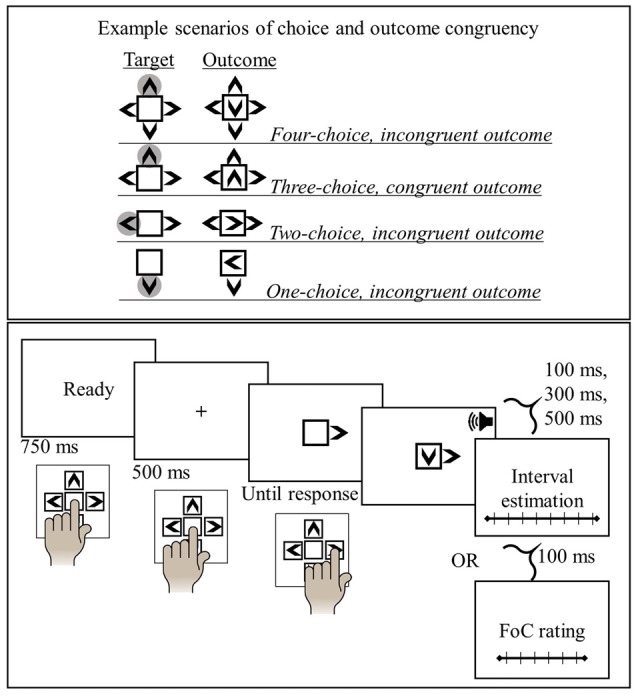
Illustration of the experimental conditions (upper panel) and procedure (lower panel) in interval estimation and feeling of control (FoC) tasks.

Target stimuli consisted of images of a blank square surrounded by arrowheads that varied in number and combination (see Figure 1). The images of arrowheads depicted the alternative actions on the response pad. For each trial, outcome visual stimuli were the same as the target stimuli except for the addition that the central square included one of four arrowheads (randomly determined for each trial). Outcome visual stimuli was presented simultaneously with a short beep sound (100 ms, 1000 Hz) that was delivered at 60 dB through the headphones.

### Procedure

Participants were first familiarized with the task and the stimuli and completed 15 practice trials. Practice session was followed by a 288-trial block of interval estimation task and a 96-trial block of FoC rating task. In each block, choice-level (one, two, three, four), outcome congruency (congruent, incongruent) and delay (100 ms, 300 ms, 500 ms; for the interval estimation task only) were presented equiprobably in a mixed and random order. After the completion of each 48 trials, the experiment paused for the participants to take a short break and participants pressed any key to resume the experiment when they were ready.

Each trial in the interval estimation session began with a 750 ms presentation of a signal “Ready!”, in response to which participants were told to rest their left index finger on the central key. A fixation cross was then presented for 500 ms and followed by the target image which remained on the screen until a key press response was given. The target image was a blank square surrounded by one-to-four arrowheads (left-, right, up- or down-pointing) depending on the choice-level in a specific trial. Except for the one-choice condition, participants were told to make a free selection among the options they were presented with. They were also advised to avoid giving stereotyped responses but regard each trial on its own in the free-choice conditions. In the one-choice condition, they had no choice but were to press the instructed key. The two- and three-choice conditions included all possible combinations selected among the four options. Participants were told to respond as fast as possible and in case of an erroneous key press that could occur in one-, two- and three-choice conditions, a warning message was presented (“Incorrect key press, please pay attention”) and the trial ended without presenting the action-outcome. A valid response was followed by the outcome stimulus presented after one of three delays (100 ms, 300 ms, 500 ms). The outcome image was presented simultaneously with a beep sound and remained on the screen for 500 ms. Participants were told that key press-tone intervals would be determined randomly in each trial and could not exceed 1000 ms. At the end of each trial, the interval estimation scale was presented on the screen and participants were to indicate their estimation of the delay between their key press and the change in the central square. They did so by moving the mouse cursor along the scale with their right hand. Inter-trial interval was set to 500 ms during which a blank screen was presented.

Trial procedure in the FoC rating block was the same as in the interval estimation block with the following exceptions. First, the action-outcome delay was set fixed at 100 ms as the influence of this delay on FoC judgments was not of interest for the current study. Second, at the end of each trial, participants rated on a 6-point Likert scale (1: the lowest level of control; 6: the highest level of control) presented on the screen to indicate the degree of control they felt over the change occurred in the central square. Again, they used their right hand to move the cursor along the scale.

At the end of the experiment, participants were debriefed about the goal of the study and compensated for their time.

### Data Processing

#### Raw Data Outlier Exclusion

Trials with RTs or interval estimations being three standard deviations away from the mean, or those with incorrect responses (pressing the wrong key in all but four-choice condition) were excluded (*M*_excluded_ = 1.76%, *SD* = 0.74% of all trials).

#### Participant Exclusion

Participant exclusion criteria were the proportion of excluded trials being greater than 20% or failing to demonstrate a significant trend of gradual increase in the estimations of 100 ms, 300 ms and 500 ms. For the latter criterion, we conducted linear trend analysis for each participant and found that two participants did not show a significant trend across the actual action-outcome intervals (Caspar et al., 2016). Data of these two participants thus were excluded from the following data analyses.

## Results

Data analyses were conducted using SPSS 24 and the significance level was set to 0.05. We reported the analysis of variance (ANOVA) tests using Greenhouse-Geisser correction where Mauchly’s test of sphericity was violated.

### Interval Estimations

Mean interval estimations for each condition are shown in Table 1. In order to examine the effects of choice-level and outcome congruency, we conducted a 4 × 2 × 3 repeated measures ANOVA with choice-level (one, two, three, four), congruency (congruent, incongruent) and delay (100 ms, 300 ms, 500 ms) as within-subjects factors. The analysis yielded significant main effects of choice-level (*F*_(3,63)_ = 6.01, *p* = 0.001, $\eta_{\text{p}}^{\text{2}}$ = 0.22), outcome congruency (*F*_(1,21)_ = 4.64, *p* = 0.047, $\eta_{\text{p}}^{\text{2}}$ = 0.17) and delay (*F*_(2,42)_ = 129.17, *p* < 0.001, $\eta_{\text{p}}^{\text{2}}$ = 0.86). Additionally, we found a significant interaction between choice-level and delay (*F*_(6,126)_ = 5.51, *p* < 0.001, $\eta_{\text{p}}^{\text{2}}$ = 0.21). The interaction between choice-level and congruency and the remaining interactions were not significant (*F*s < 1.13, *p*s > 0.349).

**Table 1:** Means and standard deviations of interval estimations (ms) in each choice-level, outcome-congruency and actual delay condition.

Delay	100 ms		300 ms		500 ms	
Outcome congruency Choice-level	Congruent	Incongruent	Congruent	Incongruent	Congruent	Incongruent
One	206 (±93)	235 (±99)	439 (±106)	433 (±106)	637 (±135)	639 (±133)
Two	233 (±104)	234 (±108)	415 (±89)	433 (±75)	596 (±137)	602 (±113)
Three	230 (±101)	238 (±107)	393 (±98)	431 (±108)	592 (±109)	590 (±130)
Four	226 (±109)	229 (±104)	402 (±102)	404 (±93)	592 (±120)	589 (±152)

**Figure 2:**
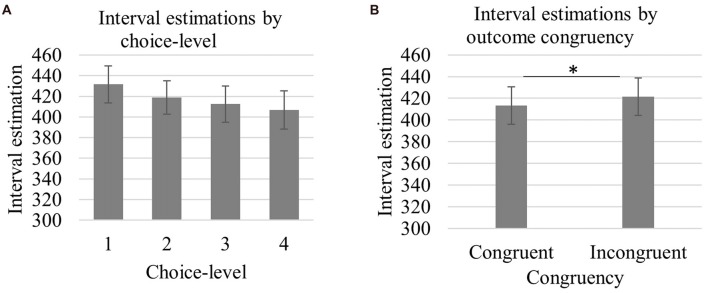
Mean interval estimations as a function of choice-level **(A)** and outcome congruency **(B)**. Interval estimations were significantly reduced with increasing choice-level (*p* < 0.001) and were greater for incongruent compared to congruent outcomes (*p = 0.047). The interval estimates were averaged across all three intervals (100 ms, 300 ms, 500 ms). Error bars represent standard error of the mean.

We were interested in whether interval estimations were reduced across one-, two, three- and four-choice conditions. We thus focused on the contrast analysis^1^ (Hervé and Williams, 2010), which indicated that there was a significant linear relationship between choice-level and interval estimations (*F*_(1,21)_ = 11.42, *p* = 0.003, $\eta_{\text{p}}^{\text{2}}$ = 0.35), such that interval estimations were reduced from one- to four-choice conditions (see Figure 2A, *M*_choice-1_ = 432, *SD*_choice-1_ = 84; *M*_choice-2_ = 419, *SD*_choice-2_ = 76; *M*_choice-3_ = 413, *SD*_choice-3_ = 82; *M*_choice-4_ = 407, *SD*_choice-4_ = 87). The main effect of outcome congruency suggested that interval estimations were shorter when outcomes were congruent (*M*_congruent_ = 413, *SD*_congruent_ = 81) compared to when they were incongruent (see Figure 2B, *M*_incongruent_ = 421, *SD*_incongruent_ = 81).

Finally, we examined the choice-level × delay interaction further by conducting repeated measures ANOVAs for each delay. Accordingly, the main effect of choice-level at 100 ms was not significant (*F* < 0.1, *p* > 0.5). At both 300 ms and 500 ms, however, the analyses yielded a significant main effect of choice-level (*F*_(3,63)_ = 5.04, *p* = 0.003, $\eta_{\text{p}}^{\text{2}}$ = 0.19; *F*_(3,63)_ = 13.47, *p* < 0.001, $\eta_{\text{p}}^{\text{2}}$ = 0.39, respectively for 300 ms and 500 ms) and contrast analysis showed that the linear trend was significant at both delays (*F*_(1,21)_ = 10.77, *p* = 0.004, $\eta_{\text{p}}^{\text{2}}$ = 0.34; *F*_(1,21)_ = 22.35, *p* < 0.001, $\eta_{\text{p}}^{\text{2}}$ = 0.39, respectively for 300 ms and 500 ms).

### FoC Ratings

**Figure 3:**
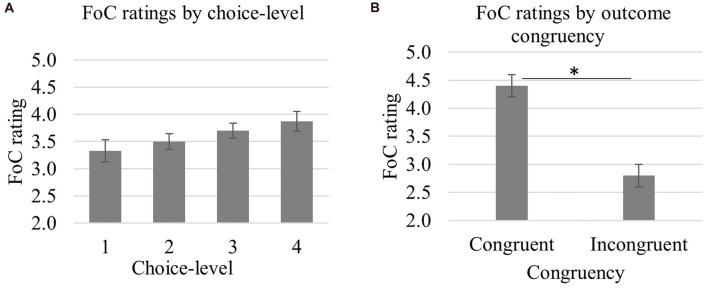
Mean FoC ratings as a function of choice-level **(A)** and outcome congruency **(B)**. FoC ratings were significantly increased with increasing choice-level (*p* = 0.027) and were higher for congruent compared to incongruent outcomes (*p < 0.001). Error bars represent standard error of the mean.

As shown in Table 2, FoC ratings were gradually increased with increased choice-level (*M*_choice-1_ = 3.32, *SD*_choice-1_ = 0.93; *M*_choice-2_ = 3.50, *SD*_choice-2_ = 0.70; *M*_choice-3_ = 3.70, *SD*_choice-3_ = 0.66; *M*_choice-4_ = 3.87, *SD*_choice-4_ = 0.84) and were higher for congruent (*M*_congruent_ = 4.40, *SD*_congruent_ = 0.94) than incongruent (*M*_incongruent_ = 2.80, *SD*_incongruent_ = 0.94) outcomes (see Figures 3A,B). We conducted a 4 × 2 repeated measures ANOVA with choice-level (one, two, three, four) and congruency (congruent, incongruent) as within subjects factors. The test revealed significant main effects of choice-level (*F*_(3,63)_ = 4.80, *p* = 0.026, $\eta_{\text{p}}^{\text{2}}$ = 0.19) and congruency (*F*_(1,21)_ = 32.88, *p* < 0.001, $\eta_{\text{p}}^{\text{2}}$ = 0.61). We also found a significant linear trend in FoC ratings with increased choice-level (*F*_(1,21)_ = 5.69, *p* = 0.027, $\eta_{\text{p}}^{\text{2}}$ = 0.21). The interaction between choice-level and congruency was not significant (*F* < 0.1, *p* > 0.7).

**Table 2:** Means and standard deviations of FoC ratings in each choice-level and outcome-congruency condition.

Outcome congruency Choice-level	Congruent	Incongruent
One	4.16 (±1.35)	2.50 (±1.18)
Two	4.24 (±0.90)	2.76 (±1.00)
Three	4.48 (±0.90)	2.92 (±0.87)
Four	4.72 (±1.21)	3.02 (±1.05)

### RTs

Mean RTs for each condition are shown in Table 3. We examined the RTs by a 4 × 4 repeated measures ANOVA with choice-level (one, two, three, four) and key (right, left, up, down) as within subjects factors. The test revealed a significant main effect of key (*F*_(3,60)_ = 3.49, *p* = 0.038, $\eta_{\text{p}}^{\text{2}}$ = 0.15) while the main effect of choice-level and the interaction between choice-level and key were non-significant (*F*s < 1.6, *p*s > 0.2). *Post hoc* multiple comparisons (Bonferroni corrected) indicated that RTs were significantly faster when pressing the right key (*M*_right_ = 506, *SD*_right_ = 137) compared to the up key (*M*_up_ = 546, *SD*_up_ = 128, *p* = 0.037). Remaining differences were not significant (*M*_left_ = 510, *SD*_left_ = 147; *M*_down_ = 526, *SD*_down_ = 126; *p*s > 0.1).

**Table 3:** Means and standard deviations of RTs (ms) in each choice-level for each key.

Key	Right	Left	Up	Down	Choice-level
One	492 (±107)	500 (±124)	527 (±95)	525 (±102)	511 (±107)
Two	505 (±125)	517 (±160)	550 (±125)	528 (±115)	525 (±131)
Three	514 (±165)	518 (±145)	553 (±126)	534 (±153)	530 (±147)
Four	514 (±151)	503 (±159)	555 (±164)	516 (±135)	522 (±152)
	506 (±137)	510 (±147)	546 (±128)	526 (±126)	**Overall**

### Key Selection Frequency in the Multiple-Choice Conditions

We calculated the mean proportions of selecting right, left, up and down keys across all but one-choice condition (*M*_right_ = 0.29, *SD*_right_ = 0.07; *M*_left_ = 0.29, *SD*_left_ = 0.10; *M*_up_ = 0.20, *SD*_up_ = 0.09; *M*_down_ = 0.22, *SD*_down_ = 0.11) and conducted six paired-samples *t*-tests (Bonferroni corrected alpha = 0.008). The tests demonstrated that none of the differences was significant (*p*s > 0.008).

## Discussion

Previous research showed that the subjective judgment of control is enhanced when observed outcomes are congruent with the expected outcomes (Sato and Yasuda, 2005; Ebert and Wegner, 2010). However, the results regarding the effect of outcome congruency on intentional binding are rather mixed. While some studies showed that binding is stronger with congruent compared to incongruent outcomes (Moore et al., 2009; Ebert and Wegner, 2010), others have reported that action-outcome congruency did not affect intentional binding (Haering and Kiesel, 2014). In the current study, we examined the effect of outcome congruency on both intentional binding and FoC judgments in a context where the action choice-level was varied from one to four and action-outcome congruency was retrospectively determined.

To begin with, we found that intentional binding and FoC were similarly enhanced as the choice-level was increased from one to four alternatives. This finding is consistent with the results of the previous studies that examined the influence of choice-level on intentional binding and FoC judgments (Barlas and Obhi, 2013; Barlas et al., 2017) and confirms the view that greater self involvement in action selection can play an important role in the SoA (Sebanz and Lackner, 2007; Haggard, 2008; Caspar et al., 2016; Barlas et al., 2018, 2017) and lends further support to the view that processes involved during action selection can prospectively influence the SoA (Chambon et al., 2014; Haggard, 2017; Sidarus et al., 2017b).

More critically, we found that congruent outcomes led to stronger intentional binding and higher FoC ratings compared to incongruent outcomes. The finding that FoC is stronger when outcomes occur congruently with their corresponding actions has been consistently demonstrated by previous research (e.g., Sato and Yasuda, 2005; Ebert and Wegner, 2010). Although the effect of outcome congruency we found on intentional binding is also in line with the findings of Moore et al. (2009) and Ebert and Wegner (2010), it stands contradictory to the other findings showing that binding is insensitive to the congruency between actions and outcomes (Desantis et al., 2012a; Haering and Kiesel, 2014). We believe that the methodological differences and the characteristics of the action-outcome associations might account for the divergent results. In the study by Haering and Kiesel (2014), for instance, the probability of an action producing a consistent outcome was 80% and thus outcome identity was highly predictable (Experiment 1) and highly congruent with the previously learned action-outcome mappings (Experiment 2). Although the authors varied this probability as 100% and 50% in separate blocks in Experiment 3, this experiment examined the effect of whether outcome identity could be predicted (100% condition) or not (50% condition) rather than the effect of the congruency between actions and outcomes as they did in Experiments 1 and 2. In the current study, however, the probability of a congruent or an incongruent outcome to occur was kept at 50% and thus the outcome congruency was unpredictable. As such, we could directly examine the effect of retrospectively determined outcome congruency on intentional binding and FoC judgments.

Another important detail in the methodology of the current study is related to the characteristic of the action-outcome associations. That is, actions in the current study were right, left, up and down key presses and the direction of these keys were visually depicted by the outcomes. These actions and outcomes thus shared a perceptual resemblance. Similarly, in Ebert and Wegner (2010), the movement of the objects on the screen was either in the same or opposite direction of the performed actions. In contrast, the study by Haering and Kiesel (2014) included colored squares or auditory tones as the action-outcomes, which could be considered as more arbitrary to their corresponding left/right key press actions. As contended by Haering and Kiesel (2014), thus, the effect of outcome congruency on binding could depend on the existence of a meaningful relationship between actions and outcomes. However, this view requires further examination to clarify, for instance, the extent of learning and experience required to establish a meaningful relationship between irrelevant actions and outcomes such that the violation of this association can affect binding.

Notwithstanding with the previously reported contradictory results, our finding regarding the effect of outcome congruency on both intentional binding and FoC can be interpreted in light of the ideomotor theory (James, 1890; Elsner and Hommel, 2001; Hommel et al., 2001, 2003; Hoffmann, 2003) and the comparator model (Frith et al., 2000). According to the ideomotor principle, the acquisition of action-outcome associations through substantial learning can lead to the bidirectional activation of actions and outcomes. That is, selection of a specific action can activate the associated outcome and perception of an outcome can activate the corresponding action representation. Although the current study did not include a learning phase for the acquisition of four action-outcome associations, the visual similarities between the representations of actions and outcomes could have led to an *automatic* action-outcome association. Thus, the representations of possible actions/outcomes observed in the target stimuli could trigger both the selection of an action and the corresponding action-outcome association. Based on this, it is reasonable to suggest that the congruency between the pre-activated action-outcome associations and the observed outcomes resulted in stronger intentional binding and FoC. Additionally, previously mentioned internal forward models that produce the anticipations towards the sensory outcomes of actions could also address the current results. In this regard, the forward models would produce, for instance, a right-pointing arrowhead once a right key press is selected. The retrospective comparison of this prediction to the actual outcome could then result in enhanced intentional binding and stronger FoC in the case of congruent compared to incongruent outcomes (Frith et al., 2000; Blakemore et al., 2002; Frith, 2005). At this point, the presumed roles of forward model and the ideomotor theory might seem quite similar in how they address the SoA. However, it should be noted that ideomotor theory focuses more strongly on the selection of an action while the comparator model emphasizes the predictions towards the outcomes once an action is selected (for an extensive discussion on this see Hommel, 2015).

A critical question we sought to investigate was whether choice-level and outcome congruency would independently or interactively influence intentional binding and FoC judgments. In contrast to our predictions, our results supported the former possibility. That is, increased choice-level enhanced both binding and FoC independent of the congruency of outcomes and congruent outcomes led to stronger binding and FoC regardless of the number of action alternatives. Previous research examining the effect of predictive and retrospective cues in the same context found that when predictive cues are absent (Moore et al., 2009) or when actions produce outcomes at a low probability (Moore and Haggard, 2008), the SoA relies more strongly on the retrospective cues. These findings are in line with the optimal cue integration view which suggests that the effects of prospective and retrospective cues on the SoA are differentially weighted depending on their reliability in a specific context (Synofzik et al., 2009; Moore and Fletcher, 2012). In a previous study, Sidarus et al. (2013) examined the effect of action selection fluency on FoC judgments when the probability of actions producing predicted effects was 67%. Additionally, actions were preceded by either compatible or incompatible primes as in the previous studies examining selection fluency effect on the FoC judgments (e.g., Wenke et al., 2010; Chambon and Haggard, 2012). Their results showed that FoC was stronger over the expected compared to unexpected outcomes and when action primes were compatible than when they were incompatible. Moreover, the interaction between outcome expectancy and prime compatibility suggested that compatible primes enhanced the FoC more strongly for unexpected than expected outcomes. And expected outcomes enhanced the FoC more strongly when primes were incompatible than when they were compatible. We had therefore predicted that in the current study, the effect of choice-level would be stronger when actions produced incongruent compared to congruent outcomes. Inconsistent with this prediction, however, we found that choice-level and outcome congruency independently influenced binding and FoC. Although for the moment we cannot determine why an interaction between these factors was not observed as predicted, our findings provide distinct evidence that choice-level as prospective cue and outcome congruency as a retrospective cue can have independent effects on intentional binding and FoC judgments. One speculation we could propose is that choice-level and outcome congruency cues provided equiprobable reliability to the measures of the SoA we employed here, since the appearance of each choice and congruency condition in the current experiment was unpredictable to the participants.

One limitation of the current study is that we did not manipulate the relatedness of the outcomes to their actions. Future studies should contrast meaningful and arbitrary outcomes in the same experimental context and examine the outcome congruency effect on intentional binding. A further potential direction for future studies could be to determine whether substantial learning and experience with arbitrarily linked actions and outcomes could mediate the effect of outcome congruency on intentional binding. Another limitation of the present study is that we obtained interval estimations and FoC ratings in separate blocks, which precluded the examination of the relationship between intentional binding and FoC ratings. Although our rationale was to avoid any potential contamination between these two measures, it remains for the future work to directly examine the link between direct and indirect measures of the SoA. Finally, an interesting question that future work should address is whether motor preparation processes and movement parameters would be influenced by the number of action alternatives and outcome predictability. Examination of the readiness potential (Shibasaki and Hallett, 2006) and electromyography in the context of selecting one of several action alternatives that produce either predicted or unpredicted outcomes could provide valuable insight into this question.

To conclude, our investigation of the effects of choice-level and outcome congruency on the SoA, measured by intentional binding and FoC judgments, provided several important findings. First, we confirmed the previous findings that both intentional binding and FoC were enhanced with increased choice-level, suggesting that the degree of self-involvement in action selection is an important contributor to the SoA. Second, we provided additional evidence that both intentional binding and FoC were stronger for congruent compared to incongruent outcomes. Moreover, we found that choice-level and outcome congruency independently influenced intentional binding and FoC judgments. We believe that these findings provide further insight into the relatively less examined questions related to the effects of concurrently present prospective and retrospective cues on the direct and indirect measures of the SoA.

## Author Contributions

ZB designed the experiment, performed the data acquisition and statistical analysis, wrote the first draft and revised the manuscript. SK contributed the design of the study and provided critical comments on the first draft. Both authors approved the submitted version of the manuscript.

## Conflict of Interest Statement

The authors declare that the research was conducted in the absence of any commercial or financial relationships that could be construed as a potential conflict of interest.

## Footnotes

^1^We set the coefficients as 3, 1, −1, −3 for one, two, three and four choice levels, respectively.
